# A Comprehensive Review About the Use of Monoclonal Antibodies in Cancer Therapy

**DOI:** 10.3390/antib14020035

**Published:** 2025-04-11

**Authors:** Angel Justiz-Vaillant, Bijay Raj Pandit, Chandrashekhar Unakal, Sehlule Vuma, Patrick Eberechi Akpaka

**Affiliations:** Department of Pathology/Microbiology & Pharmacology, The University of the West Indies, St. Augustine Campus, St. Augustine 330912, Trinidad and Tobago; angel.justiz-vaillant@uwi.edu (A.J.-V.); bijay.pandit@uwi.edu (B.R.P.); chandrashekhar.unakal@uwi.edu (C.U.); sehlule.vuma@uwi.edu (S.V.)

**Keywords:** monoclonal antibody, immune cells, solid and haematological malignancies, personalised medicine

## Abstract

Monoclonal antibodies (mAbs) targeting various pathways in cancer therapy play crucial roles in enhancing the immune system’s ability to recognise and eliminate tumour cells. These therapies are designed to either block inhibitory immune checkpoint pathways or to target specific tumour cell markers for direct destruction. Additionally, mAbs can modulate the tumour microenvironment, enhance antibody-dependent cellular cytotoxicity, and inhibit angiogenesis, further amplifying their therapeutic impact. Below is a summary of monoclonal antibodies targeting key pathways, along with their indications and mechanisms of action, which are reviewed based on therapeutic mechanisms.

## 1. Introduction

Antibodies have become a cornerstone of cancer immunotherapy, offering a targeted approach to combat tumours [1]. Monoclonal antibodies (moAbs or mAbs) are produced to recognise antigens expressed on tumour cells, enabling precise intervention with minimal impact on healthy tissues [2]. These therapies work through various mechanisms, including immune system activation, inhibition of tumour growth, and delivery of cytotoxic agents [3].

A well-established application of antibodies is immune checkpoint blockade. Antibodies targeting “Programmed Cell Death Protein-1 (PD-1), Programmed Cell Death Ligand-1 (PD-L1), and Cytotoxic T-Lymphocyte-Associated Protein 4 (CTLA-4)” restore T-cell activity, allowing the immune system to identify and destroy tumours [4]. Another advanced strategy involves antibody–drug conjugates (ADCs), which integrate the precision of antibodies with the potency of cytotoxic agents. By directing these drugs specifically to cancer cells, ADCs reduce off-target effects [5].

Bispecific antibodies, designed to engage two different antigens simultaneously, are also gaining prominence. For example, bispecific T-cell engagers (BiTEs) like blinatumomab bring lymphocyte T into close proximity to tumour cells, facilitating immune-mediated destruction [6].

Additionally, antibodies can act as carriers for radioisotopes or other therapeutic agents in targeted delivery systems. This approach, known as radioimmunotherapy, has shown promise in certain haematological malignancies [7].

Despite their success, antibody therapies are not without challenges, including high production costs, potential resistance, and immune-related side effects [8]. Research is ongoing to improve antibody design, optimise dosing regimens, and identify biomarkers to predict patient response [9].

Antibody-based immunotherapy continues to revolutionise cancer treatment, offering hope for durable responses and improved survival in both haematological and solid tumours. Its future lies in further innovation and broader application across oncology [10].

## 2. Immunomodulatory Approaches (Checkpoint Inhibitors, Bispecific Antibodies, ADCs with Immune Modulation)

### 2.1. Immune Checkpoint Pathway

The immune checkpoint pathway plays a vital role in controlling immune responses, preventing overactivity, and maintaining self-tolerance [11]. However, cancer cells exploit these mechanisms to avoid destruction by the immune system [12]. Key checkpoints, such as CTLA-4 and PD-1, serve as major regulators in this process [13]. Their activation suppresses T-cell function, allowing tumour cells to proliferate without restraint [14]. Table 1 shows indications of immune checkpoint inhibitors, along with comments and references.

#### 2.1.1. Mechanism of Action

Immune checkpoint inhibitors block the interaction between checkpoint proteins and their ligands (e.g., PD-1/PD-L1, CTLA-4/B7), restoring T-cell activity and enabling the immune system to recognise and attack tumour cells [20,21]. However, ongoing research focuses on overcoming resistance and expanding indications through combination therapies [22,23]. “CTLA-4 inhibitors (e.g., ipilimumab) block the interaction between CTLA-4 and its ligands B7-1/B7-2 on antigen-presenting cells”, facilitating T-cell activation [24].

**Figure 1:**
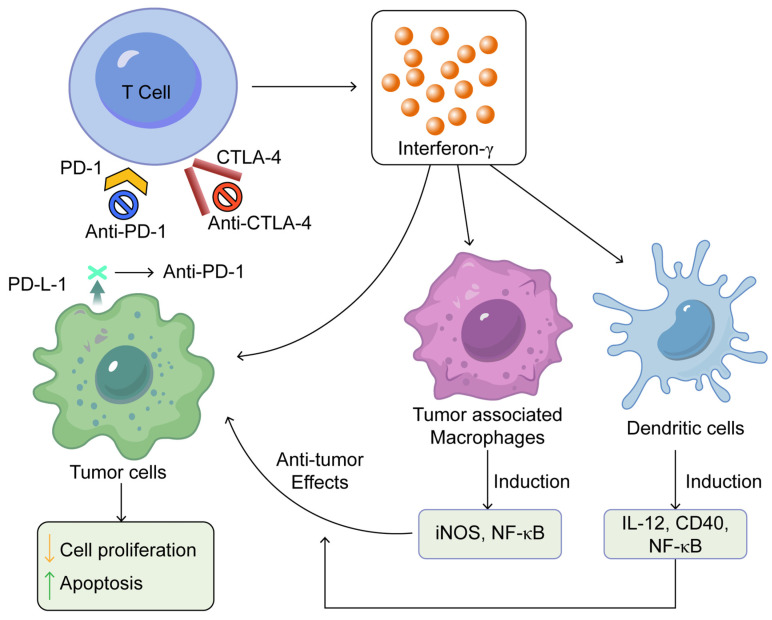
Summarises the mechanisms of action of immune checkpoint inhibitors. This was adapted from open access journal, Pandey, P. et al. *Pharmaceuticals* **2022**, *15*, 335 [25]. Adaptation performed by MDPI author services, ID: English-91110.

Immune checkpoint blockade enhances the activity of T cells that produce IFN-γ, which subsequently revitalises the antitumour capabilities of tumour-associated macrophages and dendritic cells as shown in Figure 1 [25].

#### 2.1.2. The Future of Immune Checkpoint Inhibitors

Immune checkpoint inhibitors (ICIs) have transformed oncology by enabling the immune system to effectively combat cancer. The future of these therapies lies in overcoming current limitations, expanding indications, and optimising their efficacy. Addressing tumour resistance and the variability in patient responses remains a key challenge. Research is increasingly focused on identifying biomarkers, such as tumour mutational burden (TMB) and immune profiles, to personalise treatments and predict outcomes more accurately [26].

Combination therapies hold significant potential for future cancer treatment [27]. Integrating immune checkpoint inhibitors (ICIs) with targeted therapies, chemotherapy, radiotherapy, or immunotherapies such as cancer vaccines and adoptive cell therapies aims to improve anti-tumour responses [28]. Furthermore, the advancement of next-generation ICIs targeting novel pathways, including “Lymphocyte Activation Gene-3 (LAG-3), T-cell Immunoglobulin and Mucin-Domain Containing-3 (TIM-3), and T-cell Immunoreceptor with Ig and ITIM domains (TIGIT)”, provides new opportunities for patients with treatment-resistant cancers or those unresponsive to existing therapies [29,30].

Emerging applications for ICIs are also being explored in early-stage cancers, adjuvant settings, and even non-cancerous diseases, such as infectious diseases and autoimmune disorders [31]. Advances in technology, such as artificial intelligence, are aiding in drug discovery and patient stratification [32,33], while a growing focus on reducing immune-related adverse events seeks to improve tolerability. The future of ICIs lies in broader applications, greater precision, and more durable outcomes [34,35]. Table 2 shows examples of checkpoint inhibitors and their mechanism of action.

#### 2.1.3. Use of Bispecific Antibodies: Mechanisms of Action

CD19 inhibition utilises bispecific antibodies, particularly bispecific T-cell engagers (BiTEs), to treat haematologic malignancies [45]. As a cell surface marker primarily found on B cells, CD19 serves as a key target in managing conditions such as B-cell acute lymphoblastic leukaemia (B-ALL) and non-Hodgkin lymphoma [46]. The selective targeting of CD19 has led to the development of novel therapeutic strategies in oncology.

Bispecific antibodies, for example, blinatumomab, serve a dual purpose by engaging CD19 and CD3 on malignant B-cells and T-cells, respectively. This unique mechanism facilitates the recruitment of T-cells directly to the vicinity of cancer cells, effectively bridging the gap between immune cells and tumours. Upon binding, T-cells become activated, which leads to the release of cytotoxic agents that induce apoptosis in the target tumour cells. This targeted engagement not only enhances the immune response against tumours but also minimises the impact on healthy cells, addressing a common concern associated with conventional therapies [47].

The clinical success of CD19 targeting has been evident, particularly with blinatumomab, which has achieved significant response rates in patients with relapsed or refractory B-cell malignancies. Research is also investigating the potential of CD19 inhibition in solid tumours, expanding its therapeutic applications. Blinatumomab is administered through a continuous intravenous infusion for 28 days, followed by a 14-day break. The dosage varies based on the treatment phase and patient weight. In adults, the initial dose is typically 9 mcg/day during the first week of cycle 1, increasing to 28 mcg/day in subsequent weeks and cycles [48].

#### 2.1.4. The Action of T-Cell Engagers (TCEs)

**Figure 2:**
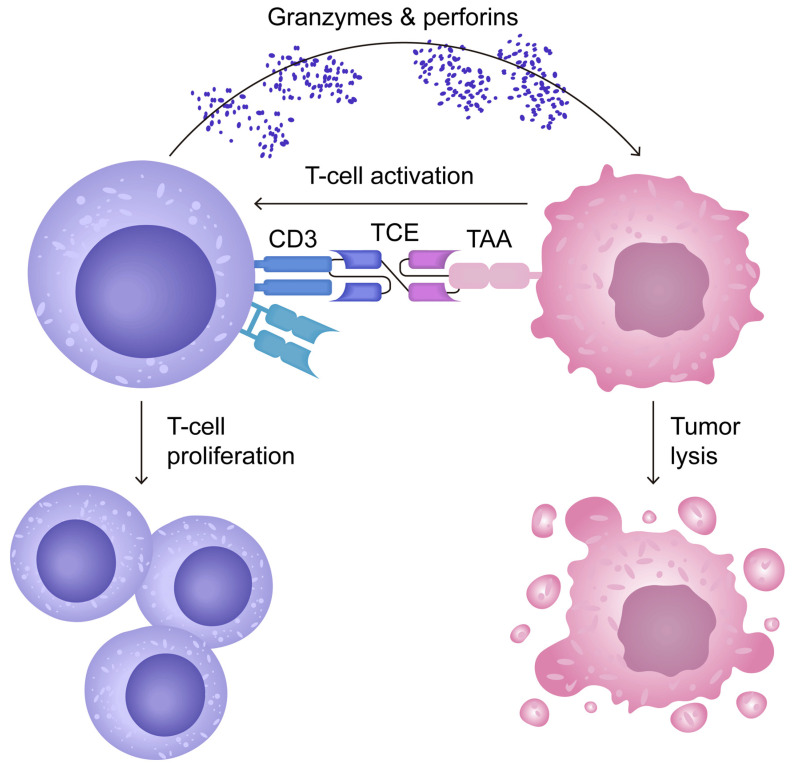
Mechanism of action of T-cell engagers (TCEs), a type of bispecific antibody. Adapted from open access journal referenced Cech, P. et al. *Cancers* **2024**, *16*, 1580 [49]. Adaptation performed by MDPI author services, ID: English-90959.

The action of T-cell engagers (TCEs), a type of bispecific antibodies, is summarised in Figure 2. It involves their dual-binding capability, as they attach to tumour-associated antigens (TAAs) and CD3 receptors. This dual interaction facilitates the establishment of a cytolytic synapse. When TCEs engage with CD3, they initiate a signal that activates T-cell cytotoxicity, leading to the release of granzymes and perforins that cause tumour cell destruction. Moreover, this engagement promotes the expansion of the T-cell population [49].

### 2.2. Antibody–Drug Conjugates (ADCs) with Immune Modulation

Antibody–drug conjugates (ADCs) with immune modulation refer to ADCs that not only deliver cytotoxic payloads to tumour cells but also enhance anti-tumour immunity [50]. These ADCs can modulate the immune response through several mechanisms:

1. Inducing Immunogenic Cell Death (ICD): Some ADC payloads, such as microtubule inhibitors (e.g., MMAE) or DNA-damaging agents (e.g., PBD dimers), can trigger ICD, leading to the release of damage-associated molecular patterns (DAMPs) that activate dendritic cells and enhance antigen presentation;
2. Targeting the Tumour Microenvironment (TME): Some ADCs are designed to deplete regulatory T cells (Tregs) or myeloid-derived suppressor cells (MDSCs), by targeting specific markers like CD25 or CCR8;
3. Enhancing T-cell Infiltration: ADCs can modify the tumour microenvironment by reducing stromal barriers or disrupting tumour-associated vasculature, thereby facilitating better immune cell infiltration;
4. Activating Innate Immunity: ADCs can engage immune cells through Fcγ receptor interactions, promoting phagocytosis or antibody-dependent cellular cytotoxicity (ADCC);
5. Combining with Immune Checkpoint Inhibitors (ICIs): ADCs that induce immune responses may synergise with ICIs (e.g., anti-PD-1/PD-L1) to enhance anti-tumour immunity.

Examples of ADCs with immune modulation include the following:

▪ Enfortumab vedotin (EV, targeting Nectin-4): Induces ICD and enhances the efficacy of checkpoint inhibitors in urothelial carcinoma.

Having explored immunomodulatory strategies that reinvigorate the host immune response, we now examine therapies targeting oncogenic receptors such as HER2 and EGFR.

## 3. Receptor-Targeted Therapies (HER2, EGFR, IGF-1R Inhibitors)

### 3.1. HER2 Inhibition and Receptor Tyrosine Kinase Signalling

HER2 (human epidermal growth factor receptor 2) is a receptor tyrosine kinase involved in regulating cell growth, survival, and differentiation [51,52]. Overexpression or amplification of HER2 is commonly observed in aggressive cancers, including gastric cancers. HER2 inhibition aims to block this pathway, halting tumour growth and promoting cancer cell death [53].

The epidermal growth factor receptor (EGFR) family also comprises HER2, which activates critical intracellular signalling pathways. Upon dimerisation with other EGFR family members, HER2 triggers cascades such as the PI3K-AKT pathway (promoting cell survival and inhibiting apoptosis), the RAS-RAF-MEK-ERK pathway (stimulating cell proliferation), and the JAK-STAT pathway (regulating transcription linked to growth) [54]. Dysregulation of these pathways drives oncogenesis in HER2-positive cancers.

Therapies targeting HER2 include monoclonal antibodies like trastuzumab, which blocks receptor dimerisation, and pertuzumab, which inhibits heterodimerisation with HER3 [55]. Tyrosine kinase inhibitors (e.g., lapatinib) block HER2’s intracellular signalling activity [56], while antibody–drug conjugates such as T-DM1 (trastuzumab emtansine) is an antibody–drug conjugate (ADC) used in cancer therapy, specifically for HER2-positive breast cancer, which deliver targeted chemotherapy [57]. However, resistance to HER2-targeted therapies remains a challenge, often driven by mutations, compensatory pathways, or phenotypic changes. Current research focuses on overcoming resistance through combination therapies, immune checkpoint inhibitors, and emerging approaches like HER2-specific chimeric antigen receptor-T (CAR-T) cell therapy [58,59].

### 3.2. Trastuzumab, Pertuzumab, and Margetuximab Are Monoclonal Antibodies Targeting HER2-Positive Cancers

Trastuzumab, developed by Genentech/Roche and approved in 1998, was the first HER2-targeting therapy. It binds to the HER2 extracellular domain, inhibiting dimerisation and activating antibody-dependent cellular cytotoxicity (ADCC). It significantly improves overall survival (OS) and progression-free survival (PFS) in HER2-positive breast and gastric cancers. Administered intravenously or subcutaneously, trastuzumab is combined with chemotherapy or pertuzumab. While revolutionary, its limitations include cardiotoxicity and resistance, addressed partly by biosimilars like Ogivri and Kanjinti, as biosimilars of trastuzumab, are used to treat HER2-positive breast and gastric cancers by inhibiting tumour growth [60]. Genentech, a biotechnology company based in the United States, is a subsidiary of Roche. It is a leader in tumour immunotherapy, advancing treatments like atezolizumab (anti-PD-L1) and combination therapies that counteract cancer immune evasion. Through biomarker-driven strategies, it enhances immuno-oncology efficacy for lung, breast, and other malignancies.

Pertuzumab, also from Genentech/Roche, was FDA-approved in 2012. It targets a different HER2 epitope, blocking HER2 dimerisation with other HER family receptors, such as HER3. Pertuzumab is most effective when combined with trastuzumab and docetaxel, as demonstrated in the CLEOPATRA trial. The CLEOPATRA trial was a phase III clinical study that investigated the effectiveness and safety of combining trastuzumab, pertuzumab, and docetaxel for treating HER2-positive metastatic breast cancer. Findings showed that this combination significantly prolonged progression-free and overall survival compared to trastuzumab and docetaxel alone, setting a new standard for first-line treatment in this cancer type. This combination further enhances OS and PFS, particularly in metastatic HER2-positive breast cancer. Pertuzumab’s primary side effects include diarrhoea and neutropenia, and its patent is set to expire in 2027 [61].

**Figure 3:**
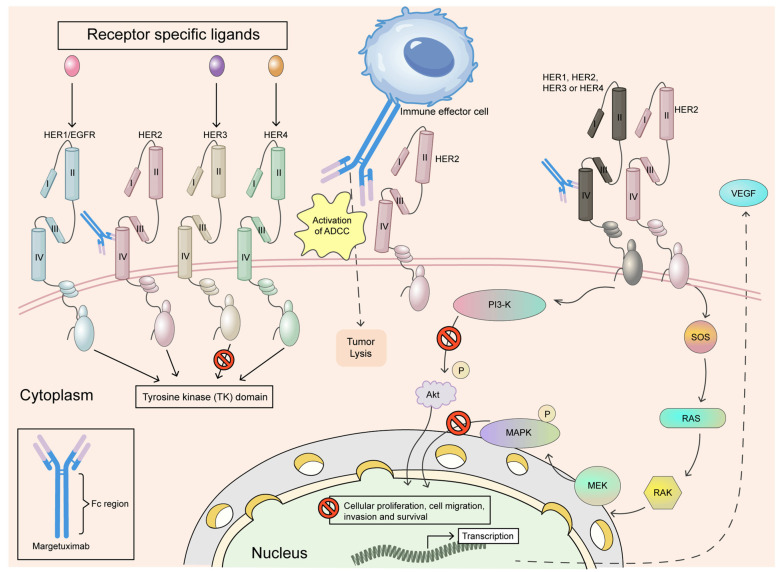
Margetuximab is a monoclonal antibody that has been modified at the Fc region to enhance its therapeutic effects. It specifically binds to the extracellular domain IV of the HER2 receptor, preventing the formation of HER2 homodimers and disrupting the ligand-independent interactions of HER2 with HER3, HER1, and HER4. This disruption strengthens antibody-dependent cellular cytotoxicity (ADCC), leading to the targeted elimination of tumour cells. Furthermore, by blocking the HER2-associated tyrosine kinase signalling pathway, margetuximab reduces cancer cell proliferation, migration, invasion, and survival. Adapted from open access journal referenced Alasmari, M.M. *Cancers* **2022**, *15*, 38 [62]. Adaptation performed by MDPI author services. ID: English-91110.

Margetuximab, developed by MacroGenics and approved in 2020, introduces an engineered Fc domain that enhances binding to activating Fc receptors, boosting ADCC as shown in Figure 3. It is indicated for HER2-positive metastatic breast cancer after prior trastuzumab regimens. The SOPHIA trial highlighted its modest efficacy improvement over trastuzumab in resistant settings. Margetuximab’s side effects include infusion-related reactions and fatigue, and it is used with chemotherapy. In addition, the SOPHIA trial was a phase III study that examined the effectiveness and safety of margetuximab in patients with HER2-positive metastatic breast cancer who had already undergone HER2-targeted treatments. It compared margetuximab plus chemotherapy with trastuzumab plus chemotherapy to assess improvements in survival outcomes. Results indicated potential benefits, particularly for individuals with the CD16A-158F allele, supporting its role as an alternative therapy [62].

Together, these drugs revolutionise HER2-targeted therapies, with trastuzumab as a cornerstone, pertuzumab offering synergistic benefits, and margetuximab enhancing immune response for resistant cases. Despite their differences, their combined use exemplifies precision oncology, advancing outcomes for HER2-positive cancer patients. In our own words, precision oncology involves tailoring treatments based on a patient’s genetic or molecular profile.

### 3.3. Epidermal Growth Factor Receptor (EGFR) in Cancer Therapy

The epidermal growth factor receptor (EGFR) plays a crucial role in inhibiting cancer cell growth and serves as a key target for treatment, particularly in malignancies characterised by EGFR overexpression or mutations. As a tyrosine kinase receptor, EGFR is integral to cellular signalling networks that regulate vital oncogenic processes, including cell growth and differentiation [63]. Genetic alterations in this pathway—such as gene amplification—are frequently associated with tumour initiation and progression. As a result, EGFR has become a significant immunotherapy target, particularly in colorectal cancer, non-small cell lung cancer (NSCLC), and head and neck squamous cell carcinoma [64].

#### 3.3.1. Mechanism of Action

EGFR signalling is triggered when ligands such as epidermal growth factor (EGF) and transforming growth factor-alpha (TGF-α) bind to its extracellular domain [65]. This binding event induces receptor dimerisation, leading to autophosphorylation of intracellular tyrosine residues and activation of downstream pathways, including PI3K/AKT and RAS/RAF/MEK/ERK [66]. These pathways drive oncogenic processes such as uncontrolled proliferation, angiogenesis, tumour invasion, and resistance to apoptosis. Aberrant EGFR signalling is closely linked to increased cancer cell aggressiveness and poorer clinical outcomes [67].

EGFR inhibitors disrupt this signalling and are classified into monoclonal antibodies and tyrosine kinase inhibitors (TKIs) [68]. Monoclonal antibodies, such as panitumumab and cetuximab, target EGFR’s extracellular domain, blocking ligand binding and preventing receptor activation [69]. By suppressing EGFR-dependent pathways, these antibodies slow tumour growth and enhance cancer cell susceptibility to treatments like chemotherapy and radiation therapy [70].

Conversely, TKIs, including gefitinib, erlotinib, and osimertinib, act on the intracellular tyrosine kinase domain of EGFR [71,72]. These small molecules competitively inhibit ATP binding, a crucial step for receptor phosphorylation and activation [73]. TKIs are especially effective in NSCLC patients with EGFR-activating mutations, such as exon 19 deletions or the L858R substitution in exon 21 [74]. These mutations increase EGFR kinase activity, making tumour cells heavily reliant on EGFR signalling for survival—a phenomenon known as “oncogene addiction” [75,76].

#### 3.3.2. Anti-EGFR Antibody Characteristics

Anti-EGFR antibodies shown in Table 3 are monoclonal antibodies targeting the EGFR, inhibiting tumour growth and proliferation. These antibodies block ligand binding, preventing downstream signalling pathways like MAPK and PI3K/AKT. Primarily used in cancers such as colorectal and head and neck, they enhance therapeutic efficacy, especially in patients with wild-type RAS genes, and are critical in precision medicine [77,78].

#### 3.3.3. FOLFIRI and FOLFOX

They are chemotherapy regimens commonly used to treat colorectal cancer. They are named based on the combination of drugs included in each regimen:

1. FOLFIRI:

FOL—Leucovorin (folinic acid);

F—5-Fluorouracil (5-FU);

IRI—Irinotecan.

FOLFIRI is often used as a first- or second-line treatment for metastatic colorectal cancer.

2. FOLFOX:

FOL—Leucovorin (folinic acid);

F—5-Fluorouracil (5-FU);

OX—Oxaliplatin.

FOLFOX is frequently used as an adjuvant therapy after surgery or as a first-line treatment for advanced colorectal cancer. Both regimens aim to inhibit cancer cell growth by targeting DNA replication and repair. The choice between FOLFIRI and FOLFOX depends on factors such as the patient’s overall health, tumour characteristics, and prior treatments [83].

## 4. VEGF/VEGFR Inhibition Cancer Therapy

### 4.1. Targeting Angiogenesis in Cancer: VEGF/VEGFR Pathway

Angiogenesis plays a fundamental role in cancer growth and progression. Tumours exploit this process to establish a blood supply, ensuring the delivery of oxygen and nutrients essential for their expansion and metastasis. The vascular endothelial growth factor (VEGF) signalling pathway, which involves VEGF and its receptor VEGFR, is a key regulator of angiogenesis [84]. The development of monoclonal antibodies (mAbs) and other inhibitors that target this pathway has led to significant advancements in cancer treatment.

### 4.2. Angiogenesis and the VEGF/VEGFR Pathway

The VEGF family comprises several ligands, including VEGF-A, VEGF-B, VEGF-C, and VEGF-D, which interact with VEGFRs—specifically VEGFR-1, VEGFR-2, and VEGFR-3—on endothelial cells. Among these, VEGF-A is the primary driver of tumour-induced angiogenesis. When VEGF-A binds to VEGFR-2, it triggers downstream signalling cascades such as PI3K/AKT and MAPK, promoting endothelial cell proliferation, migration, and the formation of new blood vessels [85]. Many tumours overexpress VEGF to accelerate angiogenesis, fostering their growth and metastatic potential [86].

Blocking VEGF/VEGFR signalling interferes with tumour vascularisation, leading to the regression of blood vessels and a decrease in the supply of oxygen and nutrients to cancerous tissues [87]. This therapeutic approach, often referred to as a “starvation” strategy, has demonstrated effectiveness in several types of cancer.

### 4.3. Indications for VEGF/VEGFR Inhibitors

A review explores the clinical effects of combining ramucirumab, a VEGFR-2 inhibitor, with docetaxel in patients with non-small cell lung cancer (NSCLC) who have previously received immunotherapy. The study evaluates the impact of this combination on tumour progression and overall survival. The findings highlight the potential of anti-angiogenic therapy alongside chemotherapy as a second-line treatment for advanced NSCLC, offering valuable insights into its efficacy and safety profile [88]. VEGF/VEGFR inhibitors are used to treat various cancers by targeting angiogenesis. Bevacizumab is approved for colorectal, lung, renal, ovarian, and glioblastoma. Tyrosine kinase inhibitors (TKIs) like sunitinib and axitinib treat renal cell carcinoma (RCC) and hepatocellular carcinoma. Ramucirumab is used for gastric, lung, and hepatocellular cancers. These inhibitors reduce tumour vascularisation, limiting oxygen and nutrient supply, thereby slowing tumour progression. Beyond oncology, they are explored for retinal diseases like age-related macular degeneration. Their efficacy is often enhanced in combination with chemotherapy or immunotherapy [89].

Under normal oxygen levels, HIF-α is hydroxylated by PHD, bound by VHL, and degraded via ubiquitination. In hypoxia, HIF-α stabilises, translocates to the nucleus, and dimerizes with HIF-β, activating genes with hypoxia-responsive elements (HREs) that regulate angiogenesis, survival, metabolism, and proliferation to adapt to low oxygen conditions.

Having described the VEGF/VEGFR inhibition cancer therapy, we now look at emerging strategies including tumour cell surface marker: Trop-2, next generation antibodies, and IgE antibodies against tumours.

## 5. Emerging Strategies (Trop-2 ADCs, Combinatorial Approaches, Next-Generation Abs)

### 5.1. Tumour Cell Surface Marker: Trop-2

Trop-2 is a protein found in high amounts on the surface of certain cancer cells, including those in breast, bladder, lung, and stomach cancers. It supports tumour growth by helping cells multiply, spread, and avoid death. This makes it a useful target for treatments that deliver toxic drugs directly to cancer cells. Blocking Trop-2 may slow disease progression, as its presence is linked to more aggressive cancers and poorer outcomes. Research continues to explore its role in therapy development.

### 5.2. Mechanism of Action: Trop-2 ADCC

Trop-2 is a surface protein highly expressed in various cancers, including breast, lung, and gastrointestinal tumours. It promotes cancer progression by enhancing cell growth, migration, and survival. Due to its role in tumour aggressiveness and poor prognosis, Trop-2 has become a promising target for antibody–drug conjugates and other therapies. Research highlights its potential for improving cancer treatment by selectively targeting malignant cells while sparing healthy tissues, making it a valuable focus for ongoing clinical development. The ADC strategy involves using monoclonal antibodies (mAbs) conjugated to cytotoxic agents. One of the prominent ADCs targeting Trop-2 is sacituzumab govitecan, approved for TNBC and urothelial carcinoma. The ADC binds specifically to Trop-2 on tumour cell surfaces, internalises into the cell, and releases the cytotoxic payload. This mechanism spares normal tissues while maximising tumour cell death, reducing off-target toxicity. SN-38, the active metabolite of irinotecan, serves as the cytotoxic payload in sacituzumab govitecan, where it interferes with DNA replication, leading to tumour cell apoptosis [90,91,92,93].

Sacituzumab govitecan (Trodelvy) is an FDA-approved antibody–drug conjugate (ADC) designed to target Trop-2, a protein that is frequently overexpressed in multiple cancers. Regulatory approval has been granted for its use in treating [94]:

▪ Metastatic triple-negative breast cancer (mTNBC): Approved in 2020 for individuals who have previously undergone at least two treatment regimens for metastatic disease;
▪ HR-positive/HER2-negative metastatic breast cancer: Approved in 2023 for patients who have received prior endocrine therapy along with at least two systemic treatments;
▪ Locally advanced or metastatic urothelial carcinoma: Approved in 2021 for patients who have been previously treated with platinum-based chemotherapy and a PD-1/PD-L1 inhibitor.

### 5.3. Other Trop-2 ADCs in Development

Datopotamab deruxtecan (Dato-DXd) is another Trop-2 ADC that utilises a topoisomerase inhibitor as its cytotoxic component, delivered through a tumour-selective, stable tetra-peptide-based cleavable linker. Datopotamab deruxtecan (brand name Datroway) was granted FDA approval on 17 January 2025, for adult patients with unresectable or metastatic hormone receptor (HR)-positive, HER2-negative breast cancer who have previously undergone endocrine therapy and chemotherapy for advanced disease [95,96].

These agents demonstrate promise across various Trop-2 overexpressing malignancies in early-phase trials. Despite advancements in Trop-2-directed ADC treatments, significant hurdles remain. These include fluctuations in Trop-2 expression, resistance development, ADC stability concerns, and the absence of reliable biomarkers to predict therapeutic success. Future studies should prioritise refining biomarker assays to accurately identify patients who will respond best to Trop-2-targeted ADCs. This could involve integrating methods like immunohistochemistry, flow cytometry, and molecular diagnostics. Establishing a clear link between Trop-2 expression levels and treatment response may aid in selecting suitable patients and adjusting dosages where necessary. Improving antibody specificity is crucial to targeting Trop-2 on cancer cells while reducing activity in healthy tissues, thereby minimising unintended toxicity. Predicting toxicity risks from combination therapies can be enhanced through gene editing techniques and 3D tumour culture models. Additionally, advanced data analytics, including machine learning, can help anticipate potential side effects based on patient-specific information. However, as with most cytotoxic treatments, the long-term effectiveness of monotherapy remains limited due to resistance development [97].

### 5.4. Next Generation Antibodies

With advancements in next-generation antibody technologies, the development of highly targeted therapies for tumours has significantly evolved. Innovations such as single-chain variable fragments, nanobodies, bispecific antibodies, Fc-engineered variants, antibody mimetics, and antibody–drug conjugates have enhanced precision in targeting tumour-specific antigens while minimising off-target effects [98].

These strategies improve therapeutic efficacy, reduce resistance, and enable combination approaches with immune checkpoint inhibitors and other modalities. The emergence of antibody biosimilars has also expanded accessibility, ensuring broader clinical applications in oncology and improving patient outcomes in various malignancies [98,99]. Monoclonal antibody development techniques include hybridoma technology, B cell immortalisation, bacterial display, yeast display, phage display, ribosome display, mammalian cell display, DNA-encoded antibodies, RNA-encoded antibodies, transgenic animal technology, single B cell technology, and artificial intelligence/machine learning [98].

Next-generation antibody technologies are transforming tumour defence, yet several emerging advancements remain underdiscussed. One promising approach is computationally designed de novo antibodies, which use AI-driven algorithms to create highly specific antibodies against tumour-associated antigens, even those with minimal immune visibility. This enables the rapid development of personalised therapies targeting unique cancer profiles. AI-driven cancer vaccines enhance precision by optimising epitope design, mRNA/DNA instructions, and patient-specific responses. Despite challenges like tumour heterogeneity and ethical concerns, AI integration promises personalised immunotherapies, advancing cancer treatment through interdisciplinary collaboration and innovation [100].

Synthetic antigen receptors (SARs) are an alternative to chimeric antigen receptors (CARs) and offer greater precision in tumour recognition, reducing off-target toxicity. Additionally, self-assembling antibody scaffolds allow for multi-target engagement, improving efficacy against tumours with heterogeneous antigen expression. This is particularly relevant in solid tumours, where immune evasion is a major challenge [101,102]. Glycoengineered antibodies are also gaining attention, as modified glycosylation patterns enhance immune activation, leading to stronger antibody-dependent cellular cytotoxicity against tumour cells [103].

### 5.5. IgE Antibodies Against Tumours

IgE immunotherapy is an emerging approach in cancer treatment that utilises FcεRI-expressing immune cells, such as mast cells, basophils, and eosinophils, to enhance immune surveillance and antibody-dependent cellular cytotoxicity (ADCC). Unlike traditional IgG-based therapies, IgE can bypass immune resistance and activate potent anti-tumour responses. A key example is MOv18 IgE, targeting folate receptor-α in ovarian cancer, which has entered clinical trials. Preclinical studies further suggest that IgE-based therapies promote long-term anti-tumour immunity. Given its tumour specificity and distinct mechanisms of action, IgE immunotherapy presents a novel and complementary strategy in oncology, warranting further research into its clinical applications. Several IgE-based therapies (e.g., MOv18 IgE targeting folate receptor-α in ovarian cancer) are currently under clinical investigation [104].

FRα is a promising target for cancer therapy due to its high tumour specificity and stability post-chemotherapy. MOv18 IgE and IMGN853 have shown early potential, though robust clinical data are lacking. Farletuzumab’s Phase III trials had mixed results, while FRα-specific CAR-T cells offer a novel approach, pending resolution of efficacy and toxicity challenges [105].

**Figure 4:**
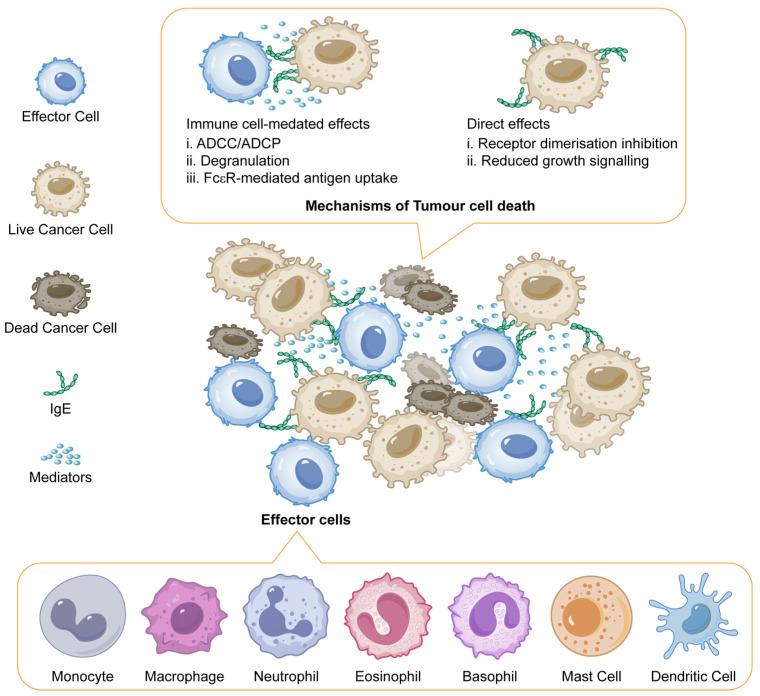
IgE mediated mechanisms of tumour cell death. Adapted from open access journal referenced Sutton, B.J. et al. *Antibodies* **2019**, *8*, 19 [107]. Adaptation made by MDPI author services. ID: English-91110.

The study examines the potential of IgE antibodies, specifically MOv18 IgE, as a more effective cancer therapy compared to traditional IgG antibodies. IgE antibodies exhibit a significantly higher affinity for Fc receptors on immune cells such as natural killer (NK) cells and macrophages, which enhances their ability to mediate immune responses against cancer cells. Preclinical studies have shown that IgE can induce superior anti-tumour activity compared to IgG in various animal models, suggesting that IgE’s unique binding properties may translate to improved clinical efficacy. To investigate the safety and efficacy of MOv18 IgE, a chimeric monoclonal IgE targeting the human folate receptor-alpha (FRα) was subjected to a Phase I clinical trial. Patients with solid tumours expressing FRα were administered escalating doses of MOv18 IgE, following skin prick tests to mitigate potential allergic reactions. The trial involved 24 patients, primarily with ovarian or endometrial cancers, who received up to six weekly infusions. The trial reported dose-limiting toxicities, including instances of urticaria and anaphylaxis at specific doses. The safety profile was deemed tolerable, with cutaneous reactions being the most common adverse events. Despite these challenges, there was evidence of anti-tumour activity among patients, with some exhibiting stable disease or responses. In summary, the trial highlights the promise of IgE therapy in cancer treatment, particularly emphasising the need for careful monitoring and patient selection to ensure safety while exploring the potential benefits of this novel treatment approach. Further studies are required to ascertain the full clinical efficacy and identify the maximum tolerated dose of MOv18 IgE [106]. IgE promotes anti-tumour immunity by activating Fc receptors on immune cells, inducing ADCC, ADCP, and degranulation, which release toxic mediators. It enhances antigen presentation and may directly inhibit cancer cell growth by blocking receptor signalling, akin to IgG antibodies (Figure 4) [107].

We explore chimeric antibodies in CAR-T cells, which enhance tumour targeting by combining the specificity of monoclonal antibodies with T-cell activation.

## 6. Integration of Monoclonal Antibodies with Chimeric Antigen Receptor (CAR) T-Cell Therapy

The development of monoclonal antibodies (mAbs) has significantly transformed cancer therapy by providing precise targeting mechanisms for various malignancies. When combined with chimeric antigen receptor (CAR) T-cell therapy, mAbs enhance immunotherapeutic effectiveness, offering tailored approaches to both haematological and solid tumour treatment [108].

Monoclonal antibodies function by specifically recognising and binding to antigens present on cancer cell surfaces. This targeted interaction either inhibits tumour growth directly or facilitates immune-mediated destruction. In CAR T-cell therapy, T cells are genetically engineered to express receptors that identify particular antigens, thereby increasing their capacity to recognise and eliminate malignant cells. This synergistic approach ultimately leads to better clinical outcomes [108,109].

For haematological malignancies such as acute lymphoblastic leukaemia (ALL) and certain lymphomas, CAR T-cell therapy has yielded exceptional results. CD19, a frequently targeted antigen, has been pivotal in these advancements [110,111]. CAR T-cell therapies like Kymriah and Yescarta, which specifically target CD19-positive cells, have demonstrated high remission rates in patients with refractory diseases, establishing them as standard treatments for select haematological cancers [112,113].

Additionally, the use of mAbs can further strengthen CAR T-cell therapy. For example, combining CAR T-cells with mAbs that inhibit co-inhibitory molecules, such as PD-1, can enhance T-cell activity and improve antitumour responses [114]. This strategy is particularly valuable in overcoming the immunosuppressive effects of the tumour microenvironment, which is a major challenge in treating solid tumours.

Solid tumour treatment with CAR T-cell therapy is complicated due to tumour heterogeneity and an immunosuppressive microenvironment that hinders T-cell efficacy [115]. While conventional mAbs have faced limitations in these settings due to antigen variability and tumour aggressiveness [116], integrating CAR technology with mAbs presents a novel strategy to improve treatment outcomes.

For solid tumours, targetable antigens include overexpressed proteins unique to specific cancer types. CAR T-cells can be designed to recognise markers such as HER2 or tumour-associated antigens (TAAs) like carcinoembryonic antigen (CEA) [117]. By leveraging mAbs in conjunction with CAR T-cells, enhanced T-cell activation can be achieved, offering a dual-targeting approach against tumours [118].

Another promising area of research involves bispecific antibodies, which can bind simultaneously to both tumour cells and T cells, promoting T-cell activation and recruitment to tumour sites [119]. These bispecific constructs, which target both the T-cell receptor and tumour-specific antigens, hold the potential for improving CAR T-cell efficacy, particularly in solid tumours.

However, challenges persist in incorporating mAbs into CAR T-cell therapies. Identifying tumour-specific antigens that ensure selective targeting without affecting normal tissues remains critical [120]. Additionally, the immunosuppressive tumour microenvironment necessitates further strategies to sustain T-cell function and improve therapeutic efficacy.

Ongoing research continues to refine these approaches. Investigators are exploring combination therapies that incorporate mAbs with checkpoint inhibitors, cytokines, and other immunotherapeutic agents. Efforts to enhance CAR construct designs and prolong T-cell persistence through genetic modifications are also being pursued [121].

**Figure 5:**
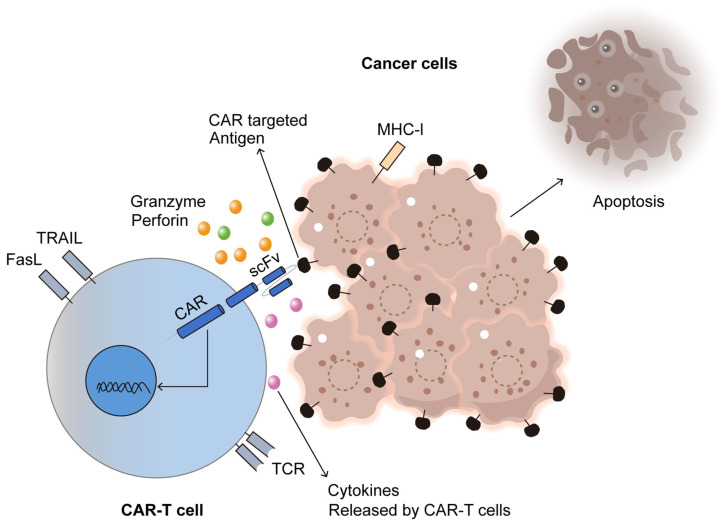
CAR-T cell immunotherapy for cancer. Adapted from Open access journal referenced by Majumder, A. *Cancers* **2023**, *16*, 39 [122]. Adaptation performed by MDPI author service. ID: English-90959.

In conclusion, integrating monoclonal antibodies with CAR T-cell therapy represents a promising advancement in cancer immunotherapy, offering solutions for both haematological and solid tumours. As research progresses, these therapies may become even more effective, leading to improved patient survival and treatment outcomes. Future clinical trials and ongoing studies will be crucial in determining the optimal approaches for harnessing the full potential of these immunotherapeutic strategies [122], as illustrated in Figure 5.

We summarise below some issues in cancer antibody immunotherapy including recent FDA approval of next-generation antibodies for cancer and we finalise with a subtopic that has brought attention in oncology: IgA and cancer.

## 7. Other Issues in Cancer Immunotherapy

### 7.1. Recent FDA Approval of Next-Generation Antibodies for Cancer

Next-generation antibodies for cancer, including bispecific and antibody–drug conjugates, have received FDA approvals, enhancing targeted therapies. These advancements improve efficacy, reduce toxicity, and offer promising treatment options for various malignancies. Recent FDA approvals of next-generation antibodies, such as immune checkpoint inhibitors and bispecific antibodies, revolutionise cancer treatment. These therapies enhance immune response, target tumour cells precisely, and improve patient overall survival with reduced side effects. These innovations not only target cancer cells with higher precision but also help overcome resistance mechanisms. FDA-approved therapies continue to evolve, providing new hope for patients with previously hard-to-treat cancers.

“Advanced, unresectable, or metastatic non–small cell lung cancer (NSCLC) harbouring a neuregulin 1 (NRG1) gene fusion with disease progression on or after prior systemic therapy. Advanced, unresectable, or metastatic pancreatic adenocarcinoma harbouring an NRG1 gene fusion with disease progression on or after prior systemic therapy”. Zenocutuzumab-zbco (Bizengri) [123] was approved by the FDA in 2024 against these tumours.

“Relapsed or refractory multiple myeloma (RRMM) in adult patients who have received at least four prior lines of therapy, including a proteasome inhibitor, an immunomodulatory agent, and an anti-CD38 monoclonal antibody”. Regarding elranatamab-bcmm (Elrexfio) [124], FDA approved in 2023 against this tumour. “Relapsed or refractory multiple myeloma (RRMM) in adult patients who have received at least four prior lines of therapy”. Talquetamab-tgvs (Talvey) [125] was approved by the FDA in 2023 against this tumour.

“Relapsed or refractory diffuse large B-cell lymphoma (DLBCL) not otherwise specified or large B-cell lymphoma (LBCL) arising from follicular lymphoma, after two or more lines of systemic therapy”. Glofitamab-gxbm (Columvi) [126] was approved by the FDA in 2023 for the treatment of this tumour.

“Relapsed or refractory diffuse large B-cell lymphoma (DLBCL) after two or more lines of systemic treatment, including DLBCL caused by indolent lymphoma and advanced B-cell lymphoma after two or more systemic treatments”. Epcoritamab-bysp (Epkinly) [127] was approved by the FDA in 2023 against this tumour.Yes

“Folate receptor alpha (FRα)-positive, platinum-resistant epithelial ovarian, fallopian tube, or primary peritoneal cancer who have received one to three prior treatments”. Mirvetuximab soravtansine (Elahere) [128] was approved by the FDA in 2022 against this tumour.

### 7.2. Exploring IgA Antibodies in Cancer Therapy: A Focus on Mucosal Surface Tumour

IgA antibodies are essential for immune defence at mucosal surfaces, including the respiratory, gastrointestinal, and urogenital tracts. They are the first line of defence against pathogens, preventing infections at these sites. Recently, IgA antibodies have gained attention in cancer immunotherapy, particularly for targeting mucosal cancers, such as those of the colon, oesophagus, and head and neck. These cancers often arise in areas rich in mucosal tissues, making IgA antibodies a promising tool for localised therapy. The potential of IgA antibodies in cancer therapy lies in their ability to recognise tumour-associated antigens present on mucosal surfaces. When these antibodies bind to such antigens, they can trigger immune responses at the tumour site, activating mechanisms such as complement activation and antibody-dependent cellular cytotoxicity (ADCC). These processes lead to the destruction of tumour cells while sparing normal, healthy tissue. This targeted approach reduces the likelihood of systemic toxicity, a common issue with many conventional cancer treatments. Researchers are currently focusing on engineering IgA antibodies to enhance their therapeutic efficacy. Advances include increasing their affinity for cancer cell antigens, improving their stability in circulation, and optimising their ability to induce immune responses specifically at mucosal sites. This targeted strategy could make IgA-based therapies particularly effective for cancers that are difficult to treat with systemic therapies alone. In addition, IgA antibodies have the advantage of being able to provide both local and systemic protection against cancer, which could result in better patient outcomes. As research in this area continues, IgA antibodies hold significant promise for improving cancer treatment, particularly in patients with mucosal cancers, offering a more specific and less toxic alternative to current therapies. The connection to immunoglobulin A (IgA) in this context could refer to the role of IgA in the mucosal immune system, particularly in the lungs. IgA is involved in the first line of defence against respiratory pathogens, including bacteria like *Chlamydia pneumoniae*. The infection may influence the immune response in lung cancer patients, potentially affecting the tumour microenvironment, immune evasion, or the body’s ability to fight cancer. Investigating this connection might offer insights into how infections like *Chlamydia pneumoniae* modulate cancer progression through immune pathways, particularly involving IgA. Another connection between immunoglobulin A (IgA) and cancer in this context might relate to IgA’s role in the mucosal immune system, which is particularly important in cancers like cervical cancer, where the tumour is often exposed to external pathogens. IgA is a key antibody in defending mucosal surfaces (such as the cervix) from infections, and its levels may change in response to immune checkpoint blockade therapy. Since ICIs can alter immune responses, this study could provide valuable insights into how IgA and other immune markers in peripheral blood might influence treatment responses, potentially serving as biomarkers for prognosis or treatment efficacy in cervical cancer patients. IgA plays a crucial role in the immunity of the respiratory and gastrointestinal tracts, where EBV is often initially encountered. The study’s focus on anti-EBV antibody responses could explore how sex differences in IgA levels or activity contribute to the immune system’s ability to control or eliminate EBV, a well-known virus directly involved in the immunopathogenesis of several cancers, including those of the head and neck. A stronger or weaker immune response could influence susceptibility to EBV-related cancers or affect the progression of existing malignancies. It could also inform how immune checkpoint blockade therapies or other immunotherapies might vary by sex in terms of efficacy, particularly in cancers associated with EBV [129,130,131,132].

### 7.3. Engineered IgA for Cancer Immunotherapy: Targeting Tumours in Mucosal Tissues

**Figure 6:**
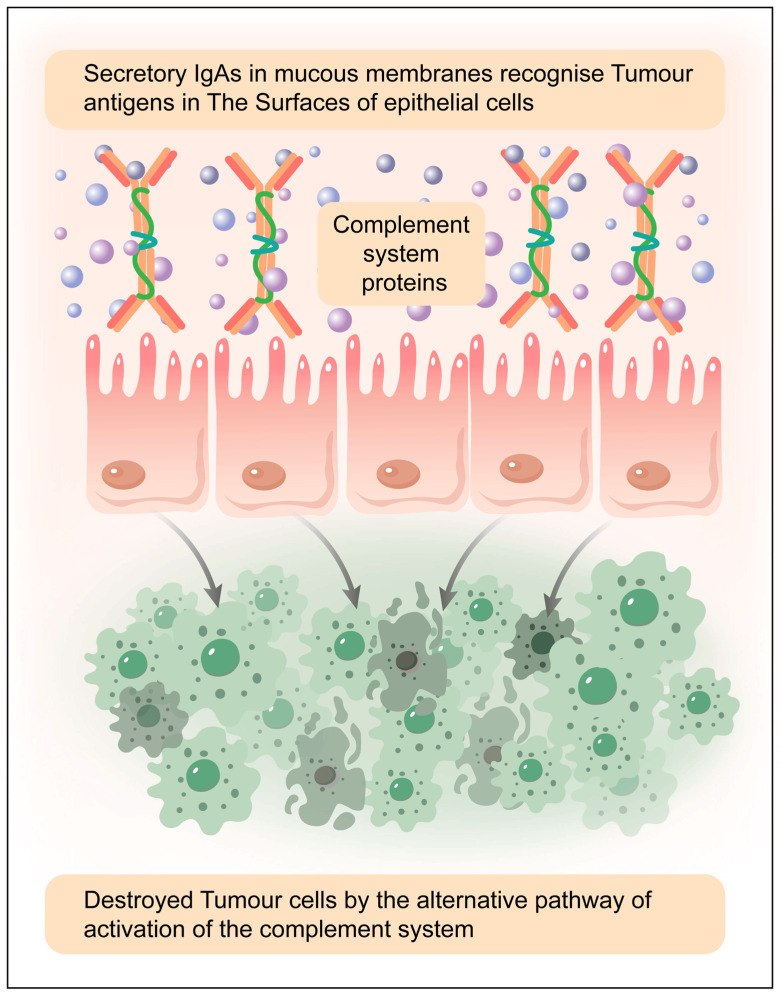
Complement activation via the alternative and lectin pathways can occur against tumour cells in mucosal secretions, but its effectiveness depends on several factors, including tumour cell surface properties, immune evasion mechanisms, the presence of secretory IgA (sIgA), and epithelial interactions. Elaborated by authors with the help of MDPI author services. MDPI author service ID: English-90959.

Secretory immunoglobulin A (IgA), the predominant antibody in mucosal secretions, can be engineered for cancer immunotherapy to target tumours in mucous membranes. Unlike IgG-based therapies, IgA naturally interacts with the polymeric immunoglobulin receptor (pIgR) and the Fc alpha receptor (FcαRI), facilitating efficient immune activation in mucosal tissues. Engineered IgA antibodies can enhance tumour cell destruction by promoting neutrophil-mediated antibody-dependent cellular cytotoxicity (ADCC) and phagocytosis. Additionally, IgA can be designed to improve stability, reduce degradation in mucosal environments, and enhance tumour specificity. Combining IgA with other immune modulators, such as checkpoint inhibitors, may further strengthen anti-tumour responses. Research suggests that IgA-based therapies could be particularly effective against mucosal malignancies, including colorectal, lung, and cervical cancers. Figure 6 shows the use of secretory IgA against tumour cells. While challenges such as manufacturing and immune regulation remain, IgA engineering offers a promising avenue for mucosal cancer immunotherapy, harnessing the body’s natural defence mechanisms in these specialised environments. IgA antibodies hold promise as anti-cancer therapeutics, but their clinical translation faces challenges due to limitations in half-life and production. Engineering strategies have improved IgA developability, achieving production levels comparable to IgG and enhancing glycan homogeneity. However, extending IgA’s serum half-life remains a challenge, with FcRn-binding sites and IgG fusion strategies showing potential. Neutrophil-engaging bispecific molecules offer promise but require further refinement. While a long half-life is beneficial, IgA’s six-day duration provides a potent, short-lived impact on tumours, positioning it between prolonged IgG exposure and small molecule therapies, expanding its potential for targeted cancer treatment [133].

The alternative pathway of the complement system is continuously active at a low level (tick-over mechanism) and can be amplified if C3b is deposited on tumour cell surfaces. However, tumour cells often express complement regulatory proteins (e.g., CD55, CD46, CD59) that prevent complement-mediated attack, reducing the effectiveness of this pathway. If these regulatory mechanisms are overcome (e.g., via therapeutic interventions or immune dysregulation), the alternative pathway can promote complement-dependent cytotoxicity (CDC) against tumour cells (Figure 6).

Tumour cells often exhibit altered glycosylation patterns, which can be recognised by mannose-binding lectin (MBL) or ficolins, leading to complement activation. The MBL-associated serine proteases (MASPs) then cleave C4 and C2, forming the C3 convertase, which can initiate downstream complement activation and contribute to tumour cell lysis or immune modulation. sIgA is not a strong activator of complement, but it can interact with epithelial cells, tumour-associated antigens, and innate immune receptors, indirectly influencing tumour cell recognition. Epithelial cells in mucosal tissues may contribute to tumour immunity by modulating complement activity and immune responses in the tumour microenvironment [134,135,136].

The potential outcomes of complement activation against tumours:

1. Direct Tumour Cell Lysis: If complement activation overcomes regulatory mechanisms, membrane attack complex (MAC) formation can lead to tumour cell death;
2. Opsonization and Phagocytosis: C3b deposition enhances recognition by immune cells, facilitating phagocytosis and antigen presentation;
3. Pro-Inflammatory Responses: Complement components (e.g., C5a, C3a) can recruit immune cells to the tumour microenvironment, promoting an anti-tumour response;
4. Immune Evasion: Many tumour cells resist complement attack by overexpressing complement inhibitors, limiting complement-mediated destruction.

## 8. Conclusions

Monoclonal antibodies (mAbs) have significantly transformed cancer therapy by providing highly specific, targeted treatment. These laboratory-engineered molecules identify and bind to antigens on cancer cells, leading to tumour inhibition through direct cytotoxicity, immune activation, and disruption of cancer-promoting pathways.

A key immune-mediated mechanism of mAbs is antibody-dependent cellular cytotoxicity, where natural killer cells bind to the Fc region of antibody-coated cancer cells, triggering apoptosis. This approach is central to therapies like rituximab for B-cell malignancies and trastuzumab for HER2-positive breast cancer. Additionally, checkpoint inhibitors such as pembrolizumab and nivolumab restore T-cell function by blocking immune checkpoint proteins, enhancing the immune response against tumours. Another category of antibody–drug conjugates like ado-trastuzumab emtansine delivers cytotoxic agents directly to cancer cells while minimising damage to healthy tissue.

Despite their success, monoclonal antibodies face challenges, including resistance due to antigen loss, tumour microenvironment modifications, or activation of alternative survival pathways. Tumour evasion strategies, such as immune checkpoint modulation and recruitment of immunosuppressive cells, also limit efficacy. Furthermore, high costs, complex production, and the need for specialised administration reduce accessibility, particularly in resource-limited settings. Immune-related adverse effects, especially with checkpoint inhibitors, can also pose risks.

To overcome these limitations, research is advancing novel strategies. Bispecific antibodies, targeting two antigens simultaneously, enhance tumour targeting and immune activation by directly linking immune cells to cancer cells. Next-generation antibody–drug conjugates with improved linker stability and potent cytotoxic payloads ensure precise tumour targeting while reducing off-target effects. Engineering Fc regions to enhance antibody-dependent cellular cytotoxicity is another approach to improve immune-mediated tumour clearance.

Combination therapies, integrating monoclonal antibodies with checkpoint inhibitors, CAR T-cell therapy, or small-molecule inhibitors, are showing promising results in improving efficacy and reducing resistance. Advances in personalised medicine enable better patient selection and tailored treatments, maximising therapeutic benefits.

Additionally, multispecific antibodies, targeting multiple pathways, are emerging as an effective approach, particularly in cancers with complex immune evasion mechanisms. Enhancing complement activity by blocking tumour-associated complement inhibitors may further strengthen immune responses, particularly in mucosal cancers. As research progresses, the integration of these approaches is expected to drive the next phase of advanced cancer therapies, offering more personalised, durable, and effective treatment options.

## Figures and Tables

**Table 1:** Immune checkpoint inhibitors, targeting pathways like PD-1, PD-L1, and CTLA-4, have emerged as effective treatments for various solid tumours.

Indication	Comments	Reference
Melanoma	These therapies revolutionised treatment by significantly improving survival rates	[15]
Non-Small Cell Lung Cancer (NSCLC)	PD-1/PD-L1 inhibitors are standard for advanced or metastatic cases	[16]
Head and Neck Cancer	Immune checkpoint inhibitors have shown efficacy in recurrent or metastatic disease	[17]
Cervical Cancer	These treatments address advanced or recurrent cervical carcinoma, particularly in PD-L1-positive patients	[18]
Urothelial Carcinoma	Effective for metastatic urothelial cancers, especially in patients who are ineligible for chemotherapy	[19]

**Table 2:** Examples of checkpoint inhibitors and mechanism of action.

Antibody	Remarks	Reference
Nivolumab	Enhances immune activity against malignant cells by blocking PD-1 on T-cells.	[36,37]
Pembrolizumab	Another PD-1 inhibitor with broad indications, including melanoma and lung cancer.	[38]
Atezolizumab	PD-L1 inhibitor used in bladder cancer and non-small cell lung cancer.	[39,40]
Avelumab	PD-L1 inhibitor, approved for Merkel cell carcinoma and urothelial carcinoma.	[41,42]
Ipilimumab	First approved CTLA-4 inhibitor, used for melanoma and in combination with PD-1 inhibitors like nivolumab for other cancers including lung and breast cancer.	[43,44]

**Table 3:** Comparison between anti-EGFR antibodies. Summary, indications, dosages, and side effects of each.

Antibodies	Remarks	Reference
Cetuximab	“Cetuximab is a chimeric monoclonal antibody that targets the epidermal growth factor receptor (EGFR), a transmembrane protein frequently overexpressed in several cancers”. By binding to EGFR, it disrupts downstream signalling pathways involved in cell proliferation, survival, and angiogenesis. Cetuximab is primarily indicated for metastatic colorectal cancer (mCRC) and squamous cell carcinoma of the head and neck (SCCHN). Indications and dosage: mCRC: Used either as monotherapy or in combination with chemotherapy regimens such as FOLFIRI or FOLFOX in patients with wild-type KRAS or NRAS tumours; SCCHN: Administered with platinum-based chemotherapy for metastatic or recurrent cases or alongside radiation therapy for locally advanced disease; Dosage: The initial loading dose is 400 mg/m^2^, followed by weekly intravenous infusions of 250 mg/m^2^. Side Effects: Common adverse reactions include an acneiform rash, hypomagnesaemia, infusion-related reactions, and diarrhoea. The presence of a skin rash is often associated with better treatment outcomes.	[79]
Panitumumab	Panitumumab is a fully human monoclonal antibody that also targets EGFR, inhibiting ligand binding and subsequent activation of cancer-promoting pathways. It is primarily employed in the treatment of *NRAS* mCRC or wild-type *KRAS*. Being fully human, panitumumab has a reduced risk of immunogenicity compared to chimeric antibodies like cetuximab. Indications and Dosages mCRC: Panitumumab is approved as monotherapy or in combination with chemotherapy regimens for patients with wild-type *RAS* tumours. The standard dosage is 6 mg/kg administered every two weeks via intravenous infusion. Side Effects Panitumumab’s adverse effects resemble those of cetuximab, including acneiform dermatitis, hypomagnesaemia, and infusion-related reactions. Rare but serious side effects include pulmonary fibrosis and severe diarrhoea.	[80,81]
Amivantamab	Amivantamab is a bispecific monoclonal antibody that simultaneously targets EGFR and MET, two proteins involved in oncogenic signalling. This dual action is particularly beneficial for tumours with EGFR exon 20 insertions, frequently observed in non-small cell lung cancer (NSCLC). In addition to inhibiting these pathways, amivantamab also triggers antibody-dependent cellular cytotoxicity (ADCC). Indications and Dosage NSCLC: Used for advanced or metastatic NSCLC with EGFR exon 20 insertion mutations, particularly after disease progression on platinum-based chemotherapy; Dosage: The initial loading dose is weight-based—1050 mg for patients under 80 kg and 1400 mg for those 80 kg or above. It is administered weekly for the first four weeks, followed by biweekly intravenous infusions. Side Effects: Common side effects include infusion-related reactions, rash, paronychia, and gastrointestinal symptoms such as nausea and diarrhoea. Infusion reactions tend to occur most frequently with initial doses, necessitating premedication with antihistamines and corticosteroids.	[82]

## Data Availability

The dataset supporting the findings of this study is included within the manuscript and its referenced sources, ensuring comprehensive access to the relevant data for further examination and analysis.
